# Preservation of glycine coordination compounds under a gamma radiation dose representative of natural mars radioactivity

**DOI:** 10.1038/s41598-022-17802-y

**Published:** 2022-08-11

**Authors:** Laura J. Bonales, Victoria Muñoz-Iglesias, Olga Prieto-Ballesteros, Eva Mateo-Martí

**Affiliations:** 1 Departamento de Evolución Molecular, Spanish Centre for Astrobiology, (CAB-CSIC), Instituto Nacional de Técnica Aeroespacial (INTA), Carretera de Ajalvir km 4, Torrejón de Ardoz, 28850 Madrid, Spain; 2 Departamento de Planetología y habitabilidad, Spanish Centre for Astrobiology, (CAB-CSIC), Instituto Nacional de Técnica Aeroespacial (INTA), Carretera de Ajalvir km 4, Torrejón de Ardoz, 28850 Madrid, Spain

**Keywords:** Planetary science, Chemistry

## Abstract

The Martian subsurface is more favorable for organic preservation than its surface because of the shielding effect of rocks from cosmic rays and UV radiation with increasing depth. Nevertheless, the natural radioactivity on Mars owing to U, Th, and K must be considered to study the possible extant and/or extinct life. Here, we demonstrate the importance of natural radiation on the amino acid glycine in two different chemical environments, GlyFeSO_4_ 5H_2_O and GlyMgSO_4_ 5H_2_O, which are coordination compounds considered relevant to Mars. The results show that after a 600 kGy dose of gamma radiation, glycine was more stable when it bonded to Mg in the GlyMgSO_4_ 5H_2_O coordination compound, it was less stable when it bonded to Fe in the GlyFeSO_4_ 5H_2_O compound. Studies on the effects of gamma radiation on preservation of organic molecules bound to minerals and other potential compounds on Mars are significantly important in the search for biosignatures.

## Introduction

The present Martian surface is dry and cold^1,2^. Moreover, owing to the lack of a global magnetic field and the low-pressure atmosphere of the red planet, its surface is exposed to high levels of ionizing radiation (IR)^3,4^ and highly oxidizing species^5^, which contribute to oxidizing materials in its surface soil.

Despite this inhospitable surface of Mars, a wide array of surface fluvial features have been observed, which combined with other signs, e.g., the widespread occurrence of clays^2^, reveal past (late Noachian to early Hesperian)^6^ warm and wet^2^ conditions that could support Earth-like life. If life ever occurred on Mars, it could have either adapted to the current hostile environment or could have disappeared. Thus, the search for present or past life must focus on places protected from harmful radiations that can destroy life and degrade biosignatures.

Regardless of the possibility that life and/or biosignatures have survived the surface radiation using different protection mechanisms, such as the natural environment and minerals to attenuate radiation^7,8^ and depositing and/or excreting UV-absorbent organic pigments or minerals^9^, the subsurface has more potential in the exploration of biological niches and/or biosignatures of extinct life^10,11^. The near-subsurface of Mars will be explored by the Rosalind Franklin rover of the ExoMars, whose main goal is to address whether life ever existed on Mars. The payload of this mission includes a drill that will retrieve subsurface samples from a maximum depth of 2 m^12^.

At several meters below the surface, natural radioactivity is the dominant form of radiation field because: (1) X-ray radiation is mostly scattered in the atmosphere, and is negligible compared to the UV radiation even at the surface^13^, (2) UV radiation is effectively absorbed in the first millimeter of any exposed rock^14^, and (3) particle radiation corresponding to the Martian surface is composed of solar energetic particles (SEP) and galactic cosmic rays (GCR)^15–17^, and has been attenuated by matter several meters below the surface. Particle radiation has been studied by calculating the absorbed dose induced by GCR, which is more relevant than sporadic SEP because it is an omnipresent radiation^18,19^. Studies have shown that the interaction of GCR matter, both atmospheric and subsurface, produces secondary neutrons that cause increase in radiation dose with increasing depth, becoming maximum at ~ 30 cm. Subsequently, the dose decreases with depth, reaching zero after several meters.

Natural radioactivity on Mars is due to the decay of long-lived isotopes of uranium thorium and potassium (^235,238^U, ^232^Th, ^40^K). Studies have calculated the dose rate and total cumulative dose over the last three million years, when Mars started drying. For example, Kminek and Bada^16^ estimated a dose of 350 μGy/year at 3 Ga, and 130 μGy/year today, from which a cumulative dose of 740 kGy was obtained for the last 3 Ga (considering the change in the dose rate over time)^20^. Pavlov *et* al.^13^ estimated a dose rate of approximately 10^−3^–10^−4^ kGy/year, by assuming isotopic ratios similar to the Martian meteorites; considering a mean value of 5 × 10^−4^ kGy/year, the total accumulated dose over a period of 3 Ga was estimated as ~ 1500 kGy.

High cumulative doses (of the order of hundreds of kGy) can degrade some organic compounds^21–23^; therefore, it is important to know the effect of these doses on the molecules of astrobiological interest, e.g., amino acids.

Amino acids have been investigated for their applicability in the detection of extinct or extant Martian microbial communities because they are the building blocks of terrestrial biochemistry and amino acid chirality helps discriminate between abiogenic and biogenic compounds^24^.

It should be noted that amino acid conservation does not depend solely on the conditions of their physical location (pressure, temperature, and UV radiation). Instead, the chemical environment around them dictates their stability. For example, minerals can mediate the effects of electromagnetic radiation by catalyzing photoreactions and protecting molecules against degradation^25^.

In this work, we studied the amino acid glycine as a target molecule in two different chemical environments, GlyFeSO_4_·5H_2_O and GlyMgSO_4_·5H_2_O. These are solid coordination compounds or complexes where glycine is bound to the metal. Their structure comprises of two cations, [M (H_2_O)_6_]^2+^ and [M (C_2_H_5_NO_2_)_2_ (H_2_O)_4_]^2+^ (M = Mg^2+^ or Fe^2+^), and two SO_4_^2−^ anions, where the glycine molecule exists in the zwitterionic form (a common chemical form of amino acid compounds in inorganic salt structures), and one oxygen atom of the carboxyl group is bonded to the central M^2+^^26,27^. These coordination compounds have been recognized as molecules of interest for planetary research, especially for Mars exploration as they can occur in the Martian soil, *i.e.,* GlyMgSO_4_·5H_2_O may form by the interaction of Martian kieserite with glycine-bearing aqueous solution^28^; similarly, GlyFeSO_4_·5H_2_O can be precipitated from the interactions of Martian Fe-sulfates and glycine aqueous solution^29^. This idea is supported by the spectral identification of Fe and Mg-bearing sulfates on the Martian surface^30–35^ by the Mars Express Observatoire pour la Minéralogie, l’Eau, les Glaces et l’Activité (OMEGA)^36^ and the Reconnaissance Orbiter Compact Reconnaissance Imaging Spectrometer for Mars (CRISM)^37^. Furthermore, chemical analysis of the Martian meteorite Nakhla showed that glycine is one of the two most predominant amino acids, with glutamic acid^38^.

Previous studies^39,40^ have shown that the chemical stability of glycine increases against photodecomposition (*i.e.,* UV radiation) when it is bonded to Mg in GlyMgSO_4_·5H_2_O. In this study, we irradiated GlyMgSO_4_·5H_2_O and GlyFeSO_4_·5H_2_O using a gamma radiation dose of 600 kGy, which is representative of the radiation dose several meters below the surface.

This paper presents the first report on the preservation of glycine under gamma radiation when chemically bound to hydrated minerals, specifically hydrated Mg- and Fe- sulfates. This study contributes to the understanding of the effect of natural radioactivity on organic molecules bound to minerals forming coordination complexes, which is relevant for the search for life.

## Results

Results of this investigation aimed to address the following questions: How much residual glycine resists the natural radioactivity of Mars? Can this amount increase or decrease when glycine is bound to Mg or Fe in molecules relevant to Mars exploration (e.g., GlyMgSO_4_·5H_2_O and GlyFeSO_4_·5H_2_O), *i.e.* can the structure of these molecules protect or increase the damage of the radiation on the glycine molecule?

Residual glycine was measured after irradiation at 600 kGy using the thermogram obtained by differential scanning calorimetry (DSC), which analyzes the decrease in the enthalpy of decomposition. This thermal analysis method has been confirmed to be suitable for investigating the effect of IR on amino acids and other molecules of biological importance^41,42^. However, this analysis did not provide any information about the identity of the fragments or degradation products of the amino acids. The issue regarding the radiation chemistry of amino acids in the solid state remains unresolved till date^43^ and is out of scope of the present study as other techniques, such as electron paramagnetic resonance spectroscopy are more suitable.

Thus, glycine, GlyMgSO_4_·5H_2_O, and GlyFeSO_4_·5H_2_O were analyzed using thermogravimetric analysis (TGA) and DSC in pristine (non-irradiated) and irradiated samples. Color changes of samples after irradiation using digital microscopy have been previously visualized, which can be related to defects forming due to irradiation damage.

### Microscopic characterization

**Figure 1:**
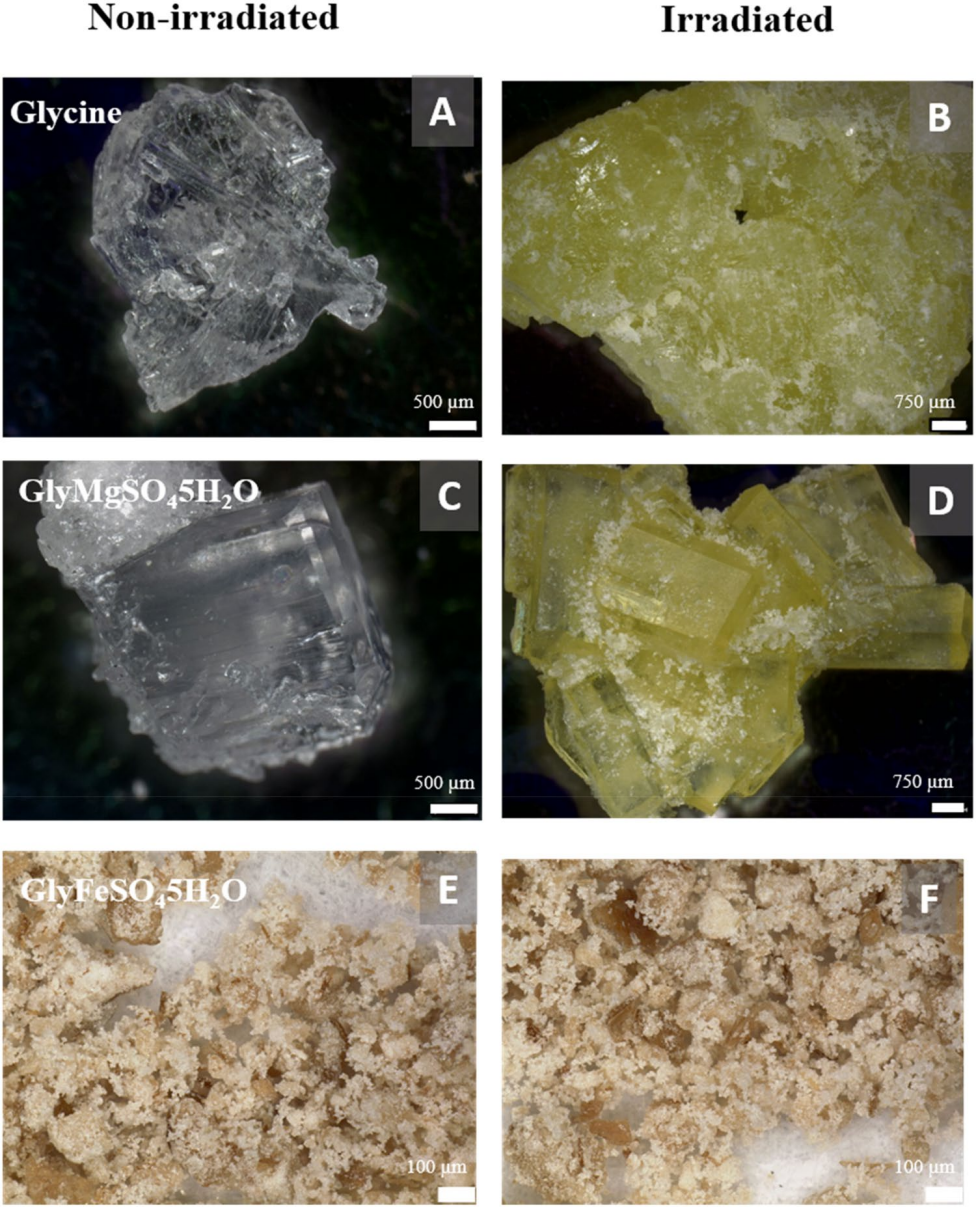
Digital images of glycine, MgSO_4_·5H_2_O, and GlyFeSO_4_·5H_2_O before (**A, C, E**) and after (**B, D, F**) 600 kGy irradiation with ^60^Co.

The color of glycine and GlyMgSO_4_·5H_2_O samples changed from transparent in the unirradiated sample to yellow at 600 kGy, but no significant color changes were observed in the GlyFeSO_4_·5H_2_O samples (Fig. 1), presumably because the initial non-irradiated sample had a color owing to the Fe^2+^ ion.

This significant darkening of the samples was attributed to the formation of color Ferbe centers (F-centers), which are crystallographic point defects produced by IR. Ionizing radiation leads to loss of electrons that become trapped in vacancies. The yellow color was the result of absorption of a photon by the trapped electron and excitation from the ground state to an excited state for the F-center^44^.

### Thermogravimetric analysis (TGA) and differential thermal analysis (DTG)

Residual amount of glycine was estimated in α-glycine, GlyMgSO_4_·5H_2_O, and GlyFeSO_4_·5H_2_O coordination compounds after 600 kGy irradiation from its enthalpy (ΔH) of decomposition, which was calculated from the thermograms obtained using DSC. To identify glycine decomposition in the DSC thermograms, we performed a TGA analysis of the irradiated and non-irradiated samples of the glycine coordination compounds. The TGA curve of α-glycine is available in the Supplementary Information (Fig. S1).

### α-Glycine

**Figure 2:**
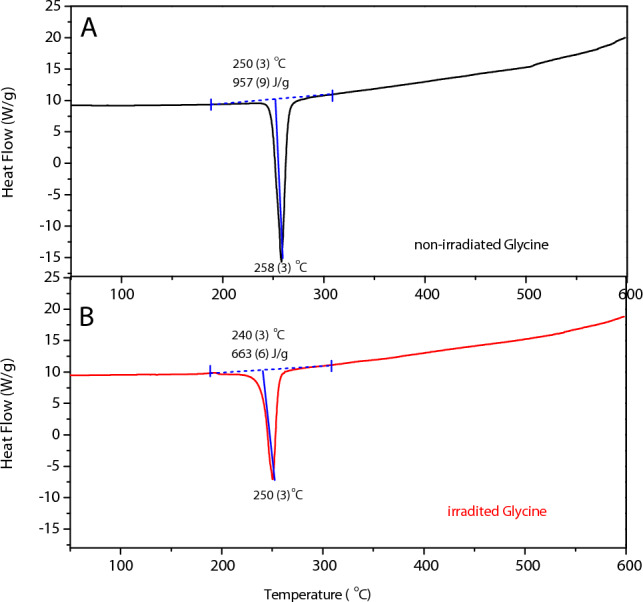
DSC curve of non-irradiated (**A**) and irradiated (**B**) glycine.

A thermogram of the pristine glycine (Fig. 2A) showed one simple endothermic peak at 257 °C corresponding to its thermal decomposition. The integrated peak yielded an enthalpy of ΔH_0_ = 956.7 J/g (*i.e.,* 71.82 kJ/mol), which was consistent with the reference value (ΔH = 72.1 kJ/mol)^45^.

Unlike the typical DSC curve of glycine, the curve for irradiated glycine showed a lower endothermic peak (ΔHγ = 49.81 kJ/mol), which shifted to a lower temperature (250 °C), (see Fig. 2B). This behavior was observed in other amino acids and related molecules after gamma radiation^37,38^, and is attributed to the reduced amino acid purity owing to the damage by IR.

The interaction of matter with IR, i.e., high-energy electromagnetic radiation (X- or gamma rays) or α- or β-particles, promotes chemical changes in solid amino acid molecules, such as the breaking of old bonds and formation of new ones. Molecular fragments are produced from radiolysis and remain trapped in the crystalline structure; they do not contribute to enthalpy as they are not part of the crystalline structure. This causes a reduction in ΔH and decomposition temperature^37^. Using Eq. (1) (see "Methods" section), the amount of residual glycine after irradiation was estimated as 69%.

### GlyMgSO_4_·5H_2_O

**Figure 3:**
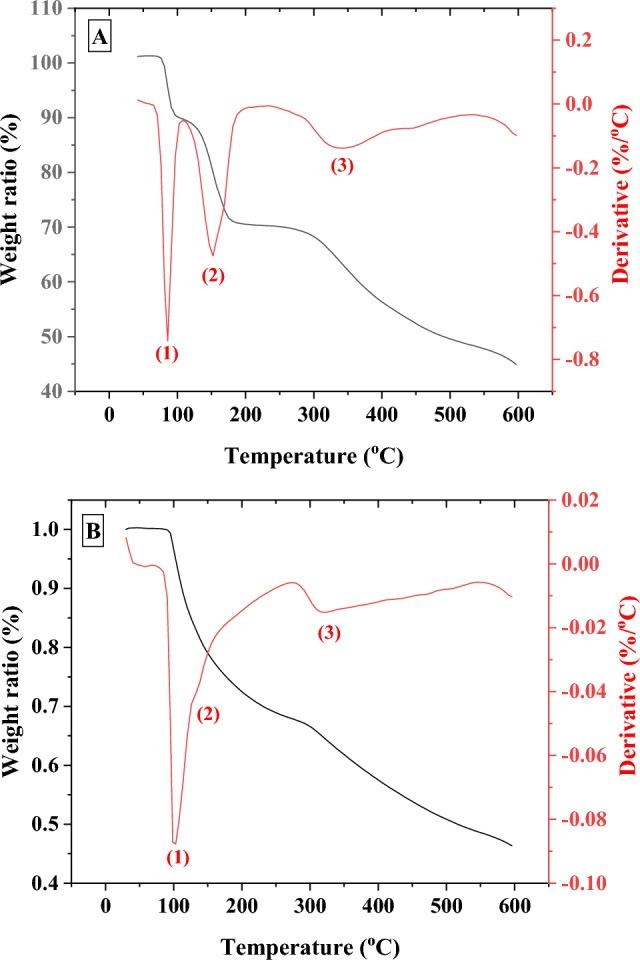
TGA and DTG curves of pristine GlyMgSO_4_·5H_2_O (**A**) and irradiated GlyMgSO_4_·5H_2_O (**B**). Black and red lines indicate TGA and DTG curves respectively.

TGA response of the non-irradiated GlyMgSO_4_·5H_2_O (Fig. 3A) showed three weight losses (Δm). If MW of the initial sample was assumed as 285.51 g/mol, the first step between 76 and 108 °C (minimum DTG at 85 °C) showed a Δm = 11.6%, which corresponds to the release of two water molecules per unit of the coordination compound (1.8 water molecules calculated, theoretical weight loss of two water molecules = 12.6%), forming the trihydrate GlyMgSO_4_ 3H_2_O^36^. In the second step, from 108 to 222 °C (minimum DTG at 152 °C), a Δm = 18.96% was obtained, which was attributed to the release of the three remaining water molecules (3.0 water molecules calculated, theoretical weight loss of three water molecules = 18.9%). The last step (Δm = 15.6%), between 273 and 434 °C (minimum DTG at 340 °C), was believed to correspond to glycine degradation. Theoretical weight loss for a complete glycine degradation was Δm = 26.29%, indicating ~ 59% of glycine to be degraded.

The TGA and DTG curves of the irradiated samples (Fig. 3B) were compared with the curves obtained for the pristine sample (Table 1). Thus, the three weight loss steps, with DTG minima curve at 98.83, 132.27, and 321 °C, can be interpreted as two losses of water molecules and one degradation of glycine, respectively. Table 1 lists the Δm values calculated from the curves displayed in Fig. 3. The calculations were made by assuming that the initial sample had a MW of 285.51 g/mol. Therefore, the results obtained for the irradiated molecule were indicative and were only used to differentiate between the coordination compound dehydration processes and glycine degradation.

**Table 1:** Calculated weight loss of pristine and irradiated GlyMgSO_4_·5H_2_O samples.

Step	Non-irradiated glycine T (°C) (± error)/Δm (wt.%) (error)/reaction	Irradiated glycine T (°C) (± error)/Δm (± wt.%) (error)/reaction assigned
1	76(1)–108 (2)/11.6 (0.2)/ release of 1.8 H_2_O molec	67(1)–106(2)/32.4(0.6) /release of 5.1 H_2_O molec
2	108(2)–222(3)/18.9(0.4)/release of 3.0 H_2_O molec	
3	220(3) 434(6)/15.6(0.3)/glycine degradation	270(3)–550(7)/glycine degradation

**Figure 4:**
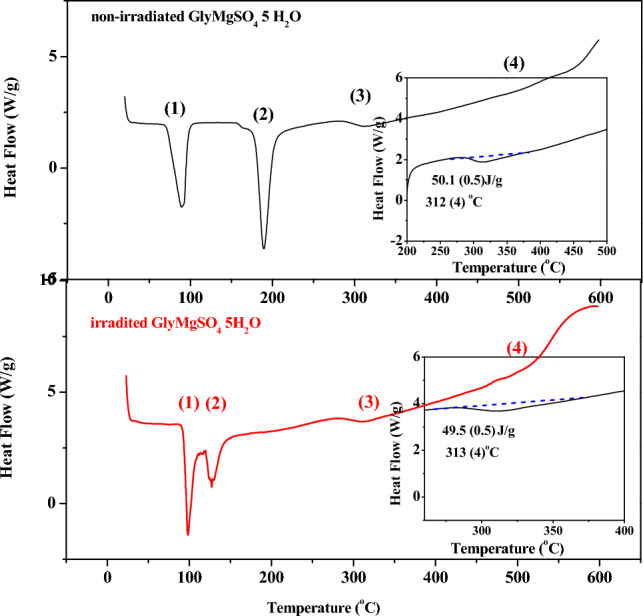
DSC curves of pristine GlyMgSO_4_·5H_2_O (**A**) and irradiated GlyMgSO_4_·5H_2_O (**B**). Insets indicate the integrated peaks corresponding to glycine degradation, which yield an enthalpy of ΔH_0_ = 50.17 J/g for the non-irradiated sample (inset of **A**) and ΔHγ = 49.54 J/g for the irradiated sample (inset of **B**).

Once glycine degradation was identified in GlyMgSO4·5H_2_O at 220–430 °C and 270–550 °C for the non-irradiated and irradiated samples, respectively, we calculated their ΔH of glycine degradation from the corresponding DSC curves (Fig. 4).

The three processes identified in the TGA can also be observed in the DSC thermogram at similar temperatures (89.99, 189.51, and 312.87 °C for non-irradiated GlyMgSO_4_·5H_2_O, and 97.99, 127.37, and 313.87 °C for irradiated GlyMgSO_4_·5H_2_O).

The integrated peaks corresponding to glycine degradation (step 3; Fig. 4) yielded an enthalpy of ΔH_0_ = 50.17 J/g (inset of Fig. 4A). Enthalpy change of the irradiated sample was ΔHγ = 49.54 J/g (inset of Fig. 4B). Thus, irradiation at 600 kGy did not degrade the sample, and the environment of glycine protected the amino acid molecule.

### GlyFeSO_4_·5H_2_O

**Figure 5:**
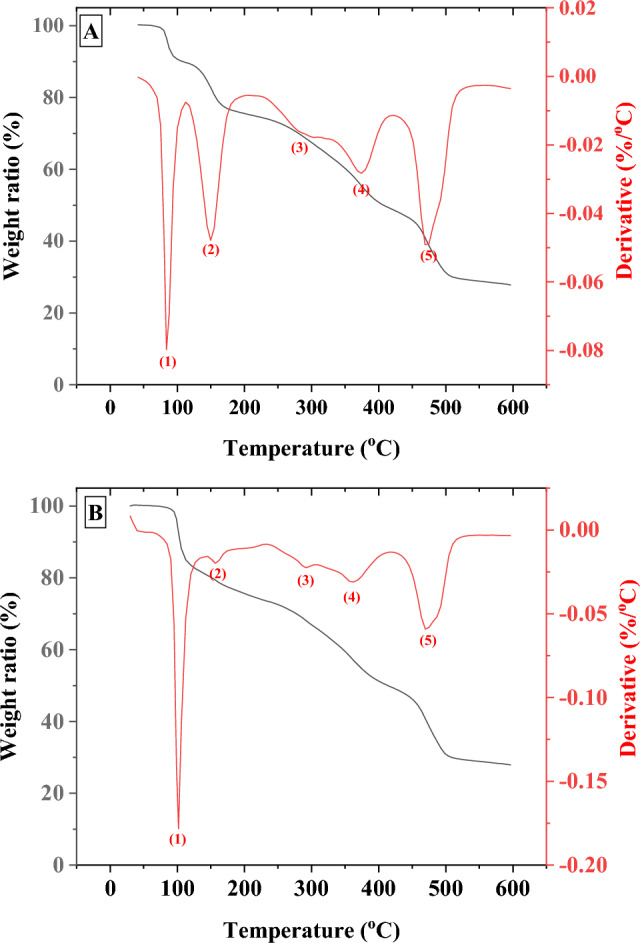
TGA and DTG curves of pristine (**A**) and irradiated (**B**) GlyFeSO_4_·5H_2_O.

**Figure 6:**
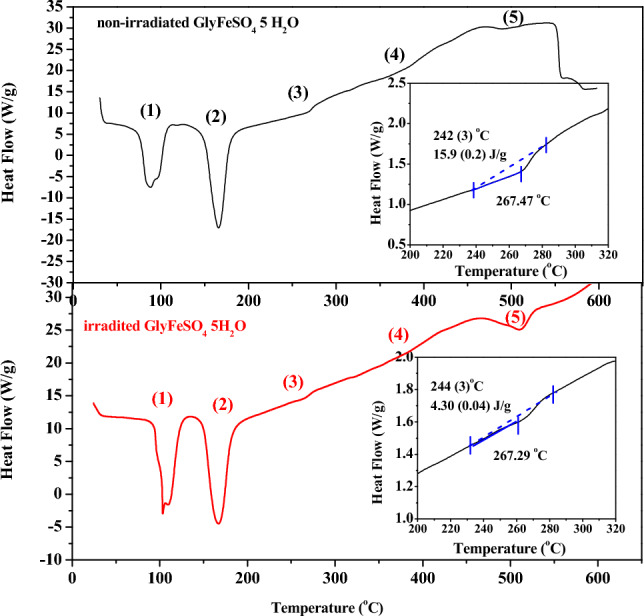
DSC curves of pristine GlyFeSO_4_·5H_2_O (**A**) and irradiated GlyFeSO_4_·5H_2_O (**B**).

Results of thermal analysis of GlyFeSO_4_·5H_2_O are shown in Figs. 5 and 6.

Several mass loss steps (Fig. 5) were detected in the TGA (black line) and DTG (red line) curves. The non-irradiated sample of GlyFeSO_4_·5H_2_O was stable up to ~ 65 °C, followed by a 10.24% weight loss from 64 to 112.5 °C, which was attributed to the release of two water molecules (expected mass loss of 11.4% for the release of two water molecules per formula unit) that formed the trihydrate phase GlyFeSO_4_ 3H_2_O. At 112–209 °C, the TGA curve exhibited a weight loss of 14.6%, which was attributed to the complete release of water (expected mass loss of 17.1% for the release of three water molecules per formula unit). Further heating of the sample led to three mass loss steps, which are associated with different reactions^46^. Based on the published data, the weight loss at 208–320 °C can be associated with a complex decomposition process of glycine, and that at 321–422 °C was associated with the oxidation of Fe^2+^ to Fe^3+^ that formed ferric sulfate (mikasaite form) and ferric oxide. Finally, the complete decomposition of sulfate ions initiated at ca. 422 °C, forming pure ferric oxide (Fe_2_O_3_, hematite) (Table 2).

**Table 2:** Mass losses (Δm) calculated from TGA for irradiated and non-irradiated GlyFeSO_4_·5H_2_O.

Step	Non-irradiated glycine T (^o^C) (± error)/Δm (± error) (wt.%)/reaction assigned	Irradiated glycine T (^o^C) (± error)//Δm (wt.%) (± error)/reaction assigned
1	64 (1)–112(2)/10.2(0.2)/release of 1.8 H_2_O molec	68(1)–139(2)/18.3(0.4)/release of 3.2 H_2_O molec
2	112(2)–209(3)/14.6(0.3)/release of 2.6 H_2_O molec	139(2)–186(3)/8.51(0.2)/release of 1.5 H_2_O molec
3	208(3)–320(5)/10.04(0.2)/glycine degradation	233(3)–327(5)/13.1(0.3)/glycine degradation
4	321(5)–422(6)/15.9(0.3)/oxidation of Fe_2_^+^	327(5)/416(6)/13.4(0.3)/oxidation of Fe_2_^+^
5	422(6)–555(8)/19.96(0.4)/decomposition of SO_4_^2-^	416(6)–545(8)/20.8(0.4)/decomposition of SO_4_^2-^

The thermogram of the irradiated sample was interpreted based on the reaction assignation performed for the non-irradiated samples (Table 2).

DSC curves of the GlyFeMgSO_4_·5H_2_O samples (Fig. 6) indicate five endothermic processes, in which the third was identified as glycine degradation by comparing with the TGA curves.

The results of enthalpy change of glycine, *i.e.*, ΔH_0_ = 15.87 J/g and ΔHγ = 4.302 J/g before and after the irradiation exposure, respectively, (inset of Fig. 6) showed that, unlike glycine stability in the Mg-based complex, glycine degrades after being irradiated at 600 kGy when it binds to Fe (GlyFeSO_4_·5H_2_O). The percentage of residual glycine (Eq. 1) was Nγ = 27.11%, *i.e.,* lesser than that when α-glycine was irradiated at sane accumulation dose.

## Discussion

The present study deals with the effect of gamma radiation on glycine preservation in two different chemical environments: GlyFeSO_4_·5H_2_O and GlyMgSO_4_·5H_2_O. These coordination compounds are considered to be relevant to Mars because they can be formed by the interaction of Martian minerals (Mg and Fe sulfates have already been identified on Mars) and glycine, with the latter being an amino acid detected in the Martian meteorite Nakhla. Additionally, the formation of these molecules requires low-pH and sulfur-rich waters, which are the inferred conditions during the Late Noachian and Hesperian on Mars^47–49^.

The cumulative radiation used in these experiments is representative of the dose at the Martian subsurface at meter-level depths, where only natural radioactivity is the predominant IR and stratigraphic analysis, using CRISM and OMEGA, suggests the occurrence of Mg- and Fe sulfates^50,51^.

For example, the sedimentary study of Mount Sharp, a ~ 5.5 km high central mound of the Gale crater by^52^ indicates mineralogical change in the stratigraphic column from polyhydrated sulfates to their monohydrated states, phyllosilicates, and finally anhydrous minerals. Specifically, polyhydrated sulfates were observed in the upper half of the height of the mound. The stratigraphy of Aram Chaos, based on CRISM data^53^, comprises monohydrated sulfate and ferric hydroxysulfate at depths of 2400–2800 m.

Moreover, the dose used in this study is consistent with scenarios where deep material has been exposed to the Martian surface for a relatively short time^54,55^. For example, recent impacts on the Martian crust can be considered a natural excavation that can provide access to samples at great depths^56^. The Kminek and Bada^16^ calculation indicates that a cumulative dose of 600 kGy at the Martian surface corresponds to 3 Mar (200 kGy/year), and 8.5 (200 kGy/year)^57^ and 1000 (0.6 kGy/year)^16^ Ma at the near subsurface (1 and 3 m under the dry regolith, respectively).

The method used to analyze the effect of IR on glycine was based on the amount of residual glycine after exposure to radiation, and the values were obtained by analyzing the DSC curves, from which the decrease in the enthalpy of decomposition was calculated.

These results provided insights into the two different behaviors for glycine when bound to different minerals. When glycine bonded with Mg in the GlyMgSO_4_·5H_2_O complex, its chemical stability against IR increased (amount of residual glycine was 100% while that of residual α-glycine after irradiation was 69%.). When glycine bonded with Fe in GlyFeSO_4_·5H_2_O, its stability decreased (amount of residual glycine was 27% and that of residual α-glycine after irradiation was 69%).

These results highlight the importance of natural radioactivity in search for biosignatures and the effect of the chemical environment of the molecules of planetological interest.

In addition to the IR stability of glycine bound to these molecules, the search for molecules of interest requires their detection by Mars rovers and landers. A common problem in this regard is identifying organic compounds in pyrolysis experiments from samples containing sulfate. For example, iron sulfates, such as jarosite, and magnesium sulfates break down to release oxygen at their pyrolysis temperatures, which is used to thermally extract organic matter, thereby representing a significant complication in organic detection^58^. In contrast, iron oxyhydroxides (goethite and hematite (Fe_2_O_3_)) do not release oxygen during thermal experiments^59^. Despite these analytical techniques, it should be noted that Raman spectroscopy, which is part of the current and future NASA and ESA planetary missions to Mars^12,60^, has proven to be a powerful technique for the characterization of these molecules under Martian conditions^39,40^^.^

## Methods

### Sample preparation

GlyMgSO_4_·5H_2_O crystals were precipitated following the procedure described in^23^, *i.e.,* glycine (Gly, NH_2_-CH_2_-COOH, purity ≥ 99%) was dissolved in Milli-Q water (total organic content < 5–10 ppm and resistivity > 18 mΩ cm^−1^) at saturation concentration; thereafter, epsomite and MgSO_4_ 7H_2_O were added at equimolar concentrations forming a solution with a pH = 5.41 ± 0.01. Slow evaporation at room temperature for two weeks yielded GlyMgSO_4_·5H_2_O crystals. FeGlySO_4_·5H_2_O was industrially produced from a hot concentrated solution with an excess of H_2_SO_4_ (low pH)^29^. It was used without any additional treatment.

### Irradiation procedure

Sample powders were irradiated at room temperature at the Náyade irradiation facility (CIEMAT)^61^, which consists of a 1.2 m^2^ × 4.5 m deep pool that uses water as the biological shield. At the bottom of the pool, 60 sources of ^60^Co (each 15 mm in diameter and 135 mm long) with a total activity of 3.22 10^14^ Bq were distributed in six sets. A cylindrical irradiation container was employed to provide a homogeneous irradiation flux within a 60 mm diameter and 100 mm volume with a ^60^Co gamma-ray source. Few tens of milligrams of α-glycine, GlyMgSO_4_·5H_2_O, and GlyFeSO_4_·5H_2_O were irradiated 600 kGy with a dose rate of 35 kGy/h, as determined by Fricke dosimetry^62^.

### Digital microscope

Morphology was analyzed using a Leica DVM6 digital microscope equipped with a motorized stage and 16:1 zoom range, covering a magnification range of 10–2, 350 ×, resolving details down to a size of 0.4 µm.

### Thermal analysis by DSC and TGA

The irradiated samples were tested for purity using DSC (TA Instruments 2920 DSC) at a heating rate of 10 °C min^−1^ under a N_2_ flow of 50 mL min^−1^ from room temperature to 600 °C. As a reference, the DSC test was also applied to the pristine (non-irradiated) samples under the same conditions. The percentage of residual glycine after solid-state radiolysis (*Nγ*) was determined from the ratio of the enthalpy after radiolysis (*ΔHγ*) to the enthalpy before radiolysis measured on the pristine sample (*ΔH*_*0*_):

1 $$N\gamma \, = \,100\left[ {\varDelta H\gamma /\varDelta H_{0} } \right]$$

following the procedure described in^41^

The TGA curves were recorded on a Mettler Toledo TGA/SDTA851e TG analyzer under nitrogen flow of 50 mL min^−1^ at a heating rate of 10 °C min^−1^ from room temperature to 600 °C using 3–5 mg of sample. The operational parameters were kept constant for all the samples to obtain comparable data.

Errors of temperature (~ 1.5%) and ΔH (~ 1%) were calculated by the difference between the true value of temperature and enthalpy of fusion of indium (156.40 °C and 28.60 J/g) and an individual measurement (158.75 °C and 28.87 J/g) at same conditions. Δm (wt.%) errors were increased by a factor of 2% based on propagation of analytical uncertainties.

### Supplementary Information

Supplementary Information.
