# Author Correction: Preservation of glycine coordination compounds under a gamma radiation dose representative of natural mars radioactivity

**DOI:** 10.1038/s41598-024-57129-4

**Published:** 2024-03-20

**Authors:** Laura J. Bonales, Victoria Muñoz-Iglesias, Olga Prieto-Ballesteros, Eva Mateo-Martí

**Affiliations:** 1 Departamento de Evolución Molecular, Spanish Centre for Astrobiology, (CAB-CSIC), Instituto Nacional de Técnica Aeroespacial (INTA), Carretera de Ajalvir km 4, Torrejón de Ardoz, 28850 Madrid, Spain; 2 Departamento de Planetología y habitabilidad, Spanish Centre for Astrobiology, (CAB-CSIC), Instituto Nacional de Técnica Aeroespacial (INTA), Carretera de Ajalvir km 4, Torrejón de Ardoz, 28850 Madrid, Spain

Correction to: *Scientific Reports* 10.1038/s41598-022-17802-y, published online 11 August 2022

The Acknowledgements section in the original version of this Article was incomplete.

“The authors used the research facilities at the Centro de Astrobiología (CAB) and were supported by the Instituto Nacional de Técnica Aeroespacial “Esteban Terradas” (INTA) projects PID2019-104205GB-C21 and PID2019-107442RB-C32, granted by the Spanish Ministerio de Ciencia, Innovación y Universidades, and by the Spanish State Research Agency (AEI) project MDM-2017-0737 granted by the Centro de Astrobiología (CSIC-INTA), Unidad de Excelencia María de Maeztu. The authors also thank Maria Gonzalez-Martinez and Maria Pilar Valles Gonzalez (Materials and Structure department of INTA) for the technical help in taking the microscopy images.”

now reads:

“The authors used the research facilities at the Centro de Astrobiología (CAB) and were supported by the Instituto Nacional de Técnica Aeroespacial “Esteban Terradas” (INTA) projects PID2019-104205GB-C21 and PID2019-107442RB-C32, granted by the Spanish Ministerio de Ciencia, Innovación y Universidades, and by the Spanish State Research Agency (AEI) project MCIN/AEI/10.13039/501100011033 MDM-2017-0737 granted by the Centro de Astrobiología (CSIC-INTA), Unidad de Excelencia María de Maeztu. The authors also thank Maria Gonzalez-Martinez and Maria Pilar Valles Gonzalez (Materials and Structure department of INTA) for the technical help in taking the microscopy images.”

The original Article has been corrected.

