# Supplementary material for: Preservation of glycine coordination compounds under a gamma radiation dose representative of natural mars radioactivity
Source: Sci Rep. 2022 Aug 11;12:13677. doi: 10.1038/s41598-022-17802-y (PMC9372174; doi:10.1038/s41598-022-17802-y)
Supplement: Supplementary file 1 — Supplementary Information. [file 41598_2022_17802_MOESM1_ESM.pdf]

# SUPPLEMENTARY INFORMATION

# Preservation of glycine coordination compounds under a gamma radiation dose representative of natural Mars radioactivity

AUTHORS NAMES AND AFFILIATIONS

Laura J. Bonales, Victoria Muñoz-Iglesias, Olga Prieto-Ballesteros, Eva Mateo-Martí

*Centro de Astrobiología (CSIC-INTA). Ctra. Ajalvir km. 4, 28850 Madrid (Spain)*

Thermogravimetry (TGA) and derived thermogravimetry (DTG) results were recorded for pristine glycine and irradiated glycine samples to investigate the processes that take place when glycine is heated at high temperature. Figures S1.A and S1.B present TG-DTG curves recorded between 40 °C and 600 °C under a nitrogen atmosphere at a heating rate of 10 ºC min$^{-1}$. The TGA curves of both samples exhibited no weight loss up to ~215 and 190 ºC for pristine glycine and irradiated glycine respectively, indicating that there is no water of crystallization or absorbed water present in the samples.

TGA curve of non irradiated glycine sample in Fig. S1.A shows three weight loss stages (two minimum in the DTA curve). The first stage with a sharp weight loss Δm = 51 % (weight ratio 48.08 %) between a temperature range of 233 °C and 320 °C, which is followed by two slow weight losing stages: the one start at 320 °C until 454 ºC corresponds to an extra weight loss Δm = 10 % (from 44.08 to 34.04 % weight ratio) and the last from 454 °C to 593 ºC with Δm =7 %. The overall weight loss measured at 600 ºC is 72.32 % (weigh ratio 27.68 %).

The thermogravimetric research of amino acids extensively studied in the last decades indicates that glycine presents, in contrast to other amino acids, a multi weight loss stages, highlighting that the analysis of the pyrolysis process of glycine is complicated. Taking

into account the results published by Li *et al.*[i] who analyzed the thermal decomposition of pure glycine by using TGA-FTIR in an inert atmosphere, the stages observed in the non-irradiated glycine TGA can be interpreted as: the first glycine decomposition in which the main gaseous products are $NH_3$, $H_2O$ and $CO_2$ due to deamination and dehydration reactions. Then, the two slow weight loss stages that form gas phase products of HNCO and CO, and HCN compound is the main gas for the higher temperatures. Figure 2.B shows that in addition to the three stages of weight loss similar to the non-irradiated glycine sample (220-320 ºC weight loss ~42 %, 320-470 weight loss ~13 % and 470 to 600 weight loss ~ 5%, Table S1), there is a first weight loss at temperatures from 173-220 ºC with a weight loss ~ 17 %, that does not appear in the corresponding non-irradiated sample. This weight loss must be due to the degradation of molecules other than pristine glycine, such as the fragments resulting from the irradiation, and therefore the gaseous products must be different, qualitatively and / or quantitatively.

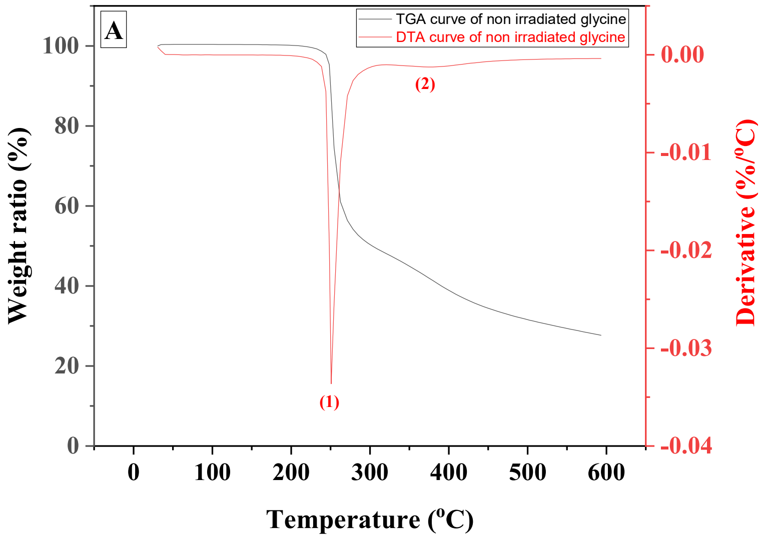

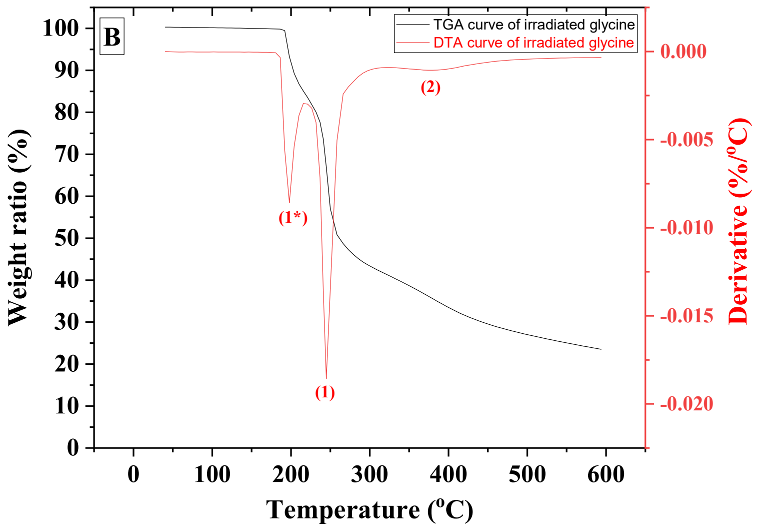


**Figure S1.** Thermogravimetric TGA and derivative DTG curve of pristine glycine (A) and irradiated glycine (B).

**Table S1.** Calculated weigh loss of pristine and irradiated $GlyMgSO_4\cdot 5H_2O$ samples

| Step | Non-irradiated $GlyMgSO_4\cdot 5H_2O$ T(oC) / Δm (wt%) | Irradiated $GlyMgSO_4\cdot 5H_2O$ T(oC) / Δm (wt%)/ |
|---|---|---|
| 1 | 233-220/ 51 % | 220-320 / 42% (173-220 / 17%) |
| 2 | 320-454 / 10 % | 320-470 /13 % |
| 3 | 454-593 / 7 % | 470-593 / 5% |

In addition, there is a shift to lower temperatures and overall a weight loss measured up to 600 °C higher for the irradiated sample, as is expected for a damage due to the ionizing radiation.

---

[i] Jie Li, Zhiyong Wang, Xi Yang, Ling Hu, Yuwen Liu, Cunxin Wang, Evaluate the pyrolysis pathway of glycine and glycylglycine by TG–FTIR, Journal of Analytical and Applied Pyrolysis, Volume 80, Issue 1, 2007, Pages 247-253, ISSN 0165-2370,
